# Distal Radius Fracture in a Surgeon's Dominant Wrist

**Published:** 2016-08-08

**Authors:** Eric Gallagher, Peter Howard, Todd Ruiter

**Affiliations:** ^a^Department of Orthopedic Surgery, Western Michigan University Homer Stryker MD School of Medicine, Kalamazoo; ^b^Borgess Medical Center, Kalamazoo, Mich

**Keywords:** distal radius fracture, modified Henry approach, utilitarian dorsal approach, occupational hand therapy, surgeon's wrist fracture

**Figure:**
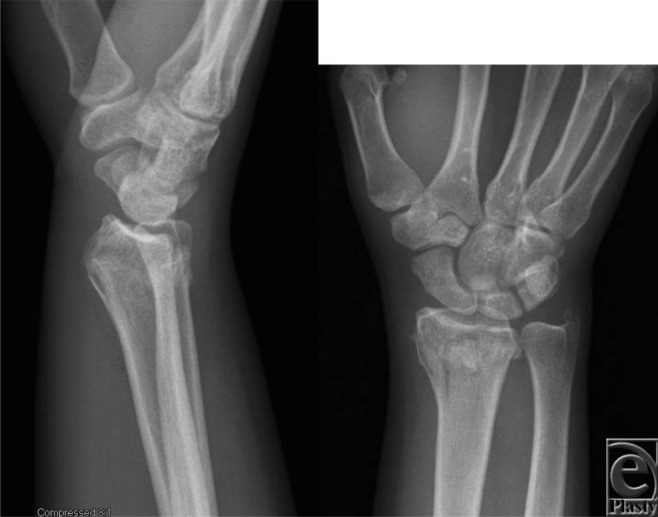

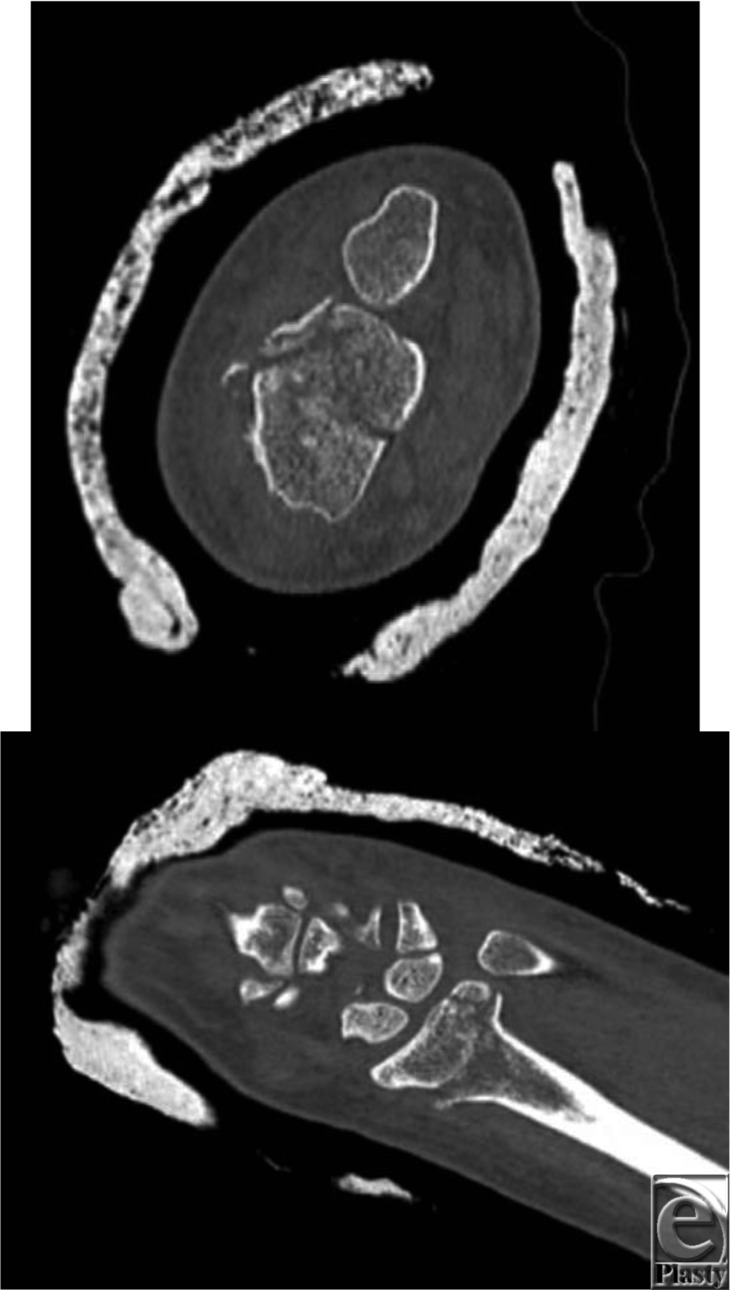

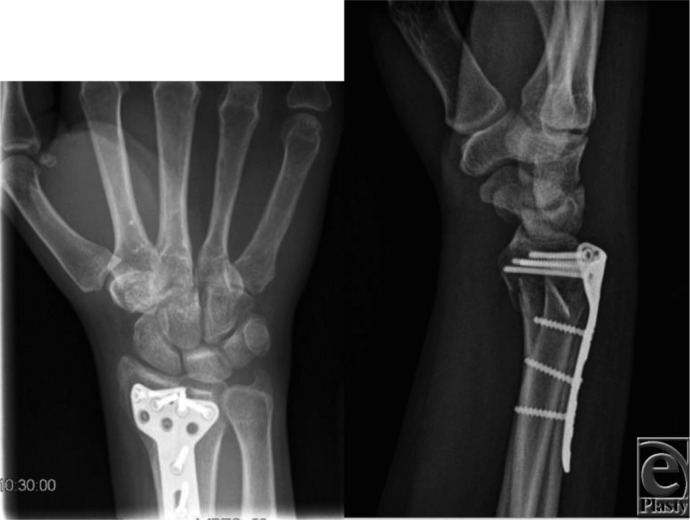


## DESCRIPTION

A 33-year-old right-hand dominant surgeon fell, sustaining a comminuted, dorsally displaced, intra-articular right distal radius fracture. Closed reduction was performed, followed by open reduction and internal fixation (ORIF) utilizing a dorsal approach. On postoperative day 6, range of motion was begun, followed by aggressive 5 days per week occupational hand therapy. She returned to her operative practice without restrictions after 7 weeks.

## QUESTIONS

1. **What are the indications for ORIF of distal radius fractures?**
2. **Describe the palmar modified Henry approach to the distal radius.**
3. **Describe the utilitarian dorsal approach to the distal radius.**
4. **Discuss hand therapy regimen after successful distal radius ORIF.**

## DISCUSSION

Indications for ORIF of distal radius fractures involve many factors including patient qualities, fracture pattern, stability, and associated injuries.^1^ Acceptable parameters for alignment are 15° or greater radial inclination, 5 mm or less shortening of radial length, less than 15° dorsal or 20° volar tilt, and less than 2 mm articular step off.^1^ Fractures that meet these parameters after reduction may be treated nonoperatively. However, they may require operative intervention if reduction is not maintained. Fractures that do not meet these parameters after reduction should undergo ORIF if the patient is an acceptable candidate for surgery. Patients who have open fractures, compartment syndrome, acute carpal tunnel, bilateral distal radius fractures, or ipsilateral proximal or distal arm fractures are generally indications for surgery.^1^ The patient's age, functional demands, and lifestyle should always factor into decision making despite fracture parameters and other injuries.

The palmar modified Henry approach begins with an approximately 8-cm longitudinal incision directly over the flexor carpi radialis (FCR).^2^^,^^3^ The FCR is retracted ulnarly to protect the median nerve.^2^ The FCR subsheath is incised, and blunt dissection is taken down to the level of the flexor pollicis longus and the pronator quadratus.^2^^,^^3^ The flexor pollicis longus is retracted either radially or ulnarly.^3^ The pronator quadratus is incised at the lateral border of the radius and elevated off the radius, leaving a cuff of pronator on the lateral aspect for repair if desired.^2^^,^^3^

The utilitarian dorsal approach begins with an incision just ulnar to Lister's tubercle, centered over the radial metaphysis.^2^^,^^3^ Incision length is based on necessary exposure for reduction and fixation; generally between 3 and 10 cm.^2^ Skin flaps are raised at the level of the extensor retinaculum.^2^ The third extensor compartment is incised, and the extensor pollicis longus is retracted radially.^3^ The fourth compartment is then elevated subperiostally to expose the distal radius.^3^ The second compartment can be elevated for further exposure if needed.^3^

After the fracture has been reduced and internally fixated, the principle of early motion should be considered.^4^ Therapy for strength and motion after surgery is commonly performed after fixation of distal radius fractures. There is no agreement on when motion should be initiated. Early motion (1–2 weeks postoperatively) has been compared with delayed motion (3–6 weeks postoperatively) in the literature. There are conflicting outcomes on early motion of the wrist after fixation. Early motion in some studies improves functional scores by 8 weeks, but equivalent outcomes for early motion versus delayed motion are seen by 3 to 6 months.^5^^-^7 There is no agreement on whether formal hand therapy should be prescribed or independent exercises should be performed at the direction of the surgeon. Availability and costs to the patient should be considered as well when deciding between formal therapy and independent therapy. Independent exercises have been shown in prospective randomized controlled studies to have greater arc of motion and strength increases at 3 and 6 months when compared with formal supervised therapy.^8^ However, it is our bias to enroll young, active patients in early motion with aggressive therapy.

Distal radius fractures are very common injuries. It is important to understand indications, approaches, and techniques for treatment in order to guide the decision-making process. As a surgeon injuring her dominant wrist, our patient's livelihood relied on a good functional outcome. After thorough discussion of the treatment options and expected outcomes, the patient elected to undergo operative treatment. Both ORIF and aggressive hand therapy helped her return to her operative practice without restrictions during the seventh postoperative week.
